# Bioavailability of Eurycomanone in Its Pure Form and in a Standardised *Eurycoma longifolia* Water Extract

**DOI:** 10.3390/pharmaceutics10030090

**Published:** 2018-06-27

**Authors:** Norzahirah Ahmad, Dodheri Syed Samiulla, Bee Ping Teh, Murizal Zainol, Nor Azlina Zolkifli, Amirrudin Muhammad, Emylyn Matom, Azlina Zulkapli, Noor Rain Abdullah, Zakiah Ismail, Ami Fazlin Syed Mohamed

**Affiliations:** 1 Herbal Medicine Research Centre, Institute for Medical Research, Jalan Pahang, Kuala Lumpur 50588, Malaysia; bpteh_km@yahoo.com (B.P.T.); murizal@imr.gov.my (M.Z.); norazlina@imr.gov.my (N.A.Z.); amiruddin@imr.gov.my (A.M.); emylynm@gmail.com (E.M.); noorrain@imr.gov.my (N.R.A.); drzakiah@gmail.com (Z.I.); ami@imr.gov.my (A.F.S.M.); 2 Aurigene Discovery Technologies Limited, Electronic City, Hosur Road, Bangalore 560100, Karmataka, India; samiulla_d@aurigene.com; 3 Medical Resource Research Centre, Institute for Medical Research, Jalan Pahang, Kuala Lumpur 50588, Malaysia; azlina@imr.gov.my

**Keywords:** eurycomanone, *Eurycoma longifolia*, bioavailability, pharmacokinetic

## Abstract

*Eurycoma longifolia* is one of the commonly consumed herbal preparations and its major chemical compound, eurycomanone, has been described to have antimalarial, antipyretic, aphrodisiac, and cytotoxic activities. Today, the consumption of *E. longifolia* is popular through the incorporation of its extract in food items, most frequently in drinks such as tea and coffee. In the current study, the characterisation of the physicochemical and pharmacokinetic (PK) attributes of eurycomanone were conducted via a series of in vitro and in vivo studies in rats and mice. The solubility and chemical stability of eurycomanone under the conditions of the gastrointestinal tract environment were determined. The permeability of eurycomanone was investigated by determining its distribution coefficient in aqueous and organic environments and its permeability using the parallel artificial membrane permeability assay system and Caco-2 cultured cells. Eurycomanone’s stability in plasma and its protein-binding ability were measured by using an equilibrium dialysis method. Its stability in liver microsomes across species (mice, rat, dog, monkey, and human) and rat liver hepatocytes was also investigated. Along with the PK evaluations of eurycomanone in mice and rats, the PK parameters for the Malaysian Standard (MS: 2409:201) standardised water extract of *E. longifolia* were also evaluated in rats. Both rodent models showed that eurycomanone in both the compound form and extract form had a half-life of 0.30 h. The differences in the bioavailability of eurycomanone in the compound form between the rats (11.8%) and mice (54.9%) suggests that the PK parameters cannot be directly extrapolated to humans. The results also suggest that eurycomanone is not readily absorbed across biological membranes. However, once absorbed, the compound is not easily metabolised (is stable), hence retaining its bioactive properties, which may be responsible for the various reported biological activities.

## 1. Introduction

Eurycomanone is uniquely found in *Eurycoma longifolia* Jack (family Simaroubaceae), which is a herbaceous tree found mainly in Southeast Asia. Eurycomanone is reported to be the most abundant phytochemical quassinoid in *E. longifolia* roots [1,2,3,4,5,6,7,8]. The MS: 2409:201 of producing a freeze-dried standardised water extract of *E. longifolia* uses eurycomanone as the chemical marker. The eurycomanone level must be consistently present at 0.80–1.50% *w*/*v*, alongside other markers such as total polysaccharides, total protein, and total glycosaponin, where their levels are expected at >20%. Traditionally, *E. longifolia* was used for ailments such as fever, wounds, and ulcers, as well as an afterbirth remedy or as a general tonic [9,10]. Biological activities of *E. longifolia* previously reported, such as male fertility enhancement [4,11] and antimalarial [12,13,14,15,16,17], cytotoxic [14,15,18], antiproliferative [19,20], and antiulcer [21] effects, are largely attributed to the quassinoids group, specifically eurycomanone.

Studies identifying eurycomanone as the compound responsible for these reported activities mainly focused on in vitro systems. Previous investigation into the physicochemical properties of several quassinoids of *E. longifolia* [16] revealed that eurycomanone and 13-*α*-(21)-epoxyeurycomanone possessed the necessary characteristics contributing to *E. longifolia*’s effect. Favourable physicochemical properties such as solubility, lipophilicity, chemical stability, permeability, plasma stability, and plasma protein binding act as indicators of the marker’s behavior in the body. The behaviour of eurycomanone in the body is of interest as the consumption of *E. longifolia* in the forms of water extracts incorporated into health supplements and beverages are the common strategies for marketed *E. longifolia*-based products. Oral consumption of *E. longifolia* in these various forms exposes its active ingredients to varying environment, which may affect their bioavailability in the body.

Bioavailability measures the delivery of an active ingredient to its site of action in order to cause the predicted effect(s). First-pass metabolism in the liver can markedly reduce the amount of eurycomanone available to the site of action, which can be predicted in vitro by assessing its metabolic stability prior to its administration orally. Previously reported eurycomanone bioavailability in rats was studied using extracts with differing amounts of eurycomanone [7,22,23].

Experimentally based data on eurycomanone’s bioavailability will be highly essential if a standardised extract is to be clinically tested. Nevertheless, to date, no bioavailability study on the standardised water extract form has been previously conducted. This study investigates the bioavailability of eurycomanone in its pure form and in a standardised water extract of *E. longifolia*.

## 2. Materials and Methods

### 2.1. Chemicals and Reagents

Standardised water extract of *E. longifolia* used conformed to the MS: 2409:201 and contained eurycomanone (1.36%), total protein (30.5%), total polysaccharide (37.8%), and glycosaponin (52%). Eurycomanone (94.8% purity) was obtained from ChromaDex Inc. (Irvine, CA, USA). Propranolol, estriol, metoprolol tartrate, diethylstil bestrol, erythromycin, atenolol, carbamazepine, propantheline bromide, enalapril, dasatinib, midazolam, terfinadine, alamethicin, uridine 5′-diphospho-glucuronosyltransferase (UDPGA), nicotinamide adenine dinucleotide phosphate (NADPH), *N*-methyl-2-pyrrolidone (NMP), 2-hydroxypropyl-beta-cyclodextrin (HPCD), and chemicals used in reagents and buffer preparations were purchased from Sigma Chemical Co. (St. Louis, MO, USA). Plasma and microsomes from mice and rats were prepared in-house, whilst plasma and microsomes from dog, monkey, and human were purchased from Thermo Fisher Scientific (Waltham, MA, USA). Solvents such as methanol, formic acid, acetonitrile (ACN), and DMSO used were of LC-MS and HPLC grade and were purchased from Fisher Chemicals (Waltham, MA, USA).

### 2.2. Animal Experiments

#### 2.2.1. Eurycomanone Assessment

The ethical approval for the animal experiments conducted in India was granted from the Animal Ethics Committee, Aurigene Discovery Technologies, Bangalore, India. Eight male Wistar rats (12 weeks old; weighing 250–350 g) and eight Caeserean Derived-1 (CD-1) mice (weighing 20–30 g) were obtained from the Animal House, Aurigene Discovery Technologies, Bangalore, India. They were supplied with standard rodent diets and drinking water ad libitum. Each rodent species (i.e., rats and mice) were divided into two groups. One group (*n* = 3 for each rodent species) was administered with eurycomanone via the intravenous route (IV) and another group (*n* = 3 for each rodent species) via the oral route (PO) using oral gavage intubation needle. Two animals of each of the species were used as a control and they received reverse osmosis water. All rats underwent jugular vein cannulation surgery 72 h prior to the administration of the eurycomanone.

#### 2.2.2. Standardised Water Extract of *E. longifolia* (SWE) Assessment

The animal experiment conducted in Malaysia was approved by the Animal Care and Use Committee, Ministry of Health Malaysia (ACUC Number: ACUC/KKM/02(1/2014)). Ten male Sprague Dawley rats (12 weeks old; weighing 250–400 g) were obtained from the Laboratory Animal Resource Unit, Institute for Medical Research, Kuala Lumpur, Malaysia. They were supplied with standard rodent diets and drinking water ad libitum. One group (*n* = 4) was administered with SWE via the IV route and another group (*n* = 4) via the PO route using oral gavage intubation needle. Two rats were used as the control and they were administered reverse osmosis water. All rats underwent jugular vein cannulation surgery 72 h prior to the administration of SWE. All animals were maintained in a 12-h light and dark cycle, the temperature was maintained between 22 ± 3 °C, and the relative humidity between 50–65%.

### 2.3. Sample Preparation

Eurycomanone (rats: 1.5 mg/mL for IV route and 3.0 mg/mL for PO route; mice: 0.5 mg/mL for IV route and 1.0 mg/mL for PO route) samples were prepared fresh on the day of dosing. For the IV administration, eurycomanone was dissolved in 3% NMP and 97% of 10% HPCD in saline. For the oral administration, eurycomanone was dissolved in 3% NMP and 97% of 30% HPCD in saline. Both samples were sonicated before use. The SWE (5 mg/mL for IV route, 10 mg/mL for PO route) was prepared fresh on the day of dosing. For the IV administration, the SWE was dissolved in reverse osmosis water and filtered (0.2 µm pore size) before administration. The SWE for the PO administration was prepared using the same method, except without filtering. The animals were not fasted and doses given were based on the body weights prior to dosing. 

### 2.4. Specimen Collection

Blood specimens (approximately 0.30–0.40 mL via the jugular vein cannula for rats and 0.10–0.20 mL via submanibular bleeding for mice) were collected at time points 0.083 (IV group only), 0.25, 0.5, 1, 2, 4, 6, and 8 h post-dosing with eurycomanone. At the experiment end point, the animals were euthanized by excess isoflurane inhalation followed by cervical dislocation. In the experiment using SWE, the rats were placed in metabolic cages (Techniplast, West Chester, PA, USA) and blood (approximately 0.30–0.40 mL collected via the jugular vein cannula), urine, and faeces specimens were collected at the same time points with the above plus one additional time point of 24 h. Kidney and liver specimens were collected at necropsy, where the rats were euthanized by CO_2_ inhalation followed by cervical dislocation. All blood specimens were centrifuged (10,000 rpm, 10 min) to obtain plasma and were stored at −80 °C until processed. Urine and faeces specimens were collected into plastic containers on wet ice while the organ specimens were snap-frozen in liquid nitrogen and maintained at −80 °C until processed.

### 2.5. Specimen Preparation for LC-MS/MS Analysis

Plasma specimens (100 µL), collected from the eurycomanone experiment were added with the internal standard solution (10 µL). Plasma proteins were precipitated with ACN (300 µL), vortex-mixed for 5 min, and centrifuged at 4000 rpm for 7 min. The supernatant was collected and evaporated using a nitrogen evaporator for 20 min at 50 °C. Thereafter, they were reconstituted with the mobile phase (500 µL) and transferred into LC-MS vials for analysis.

Plasma specimens (200 µL) from the SWE experiment were added with the internal standard solution (50 µL). Plasma proteins were precipitated with ACN (400 µL), vortex-mixed for 5 min, and centrifuged at 4000 rpm for 7 min. The supernatant was collected and evaporated to dryness using nitrogen evaporator at 60 °C. Thereafter, they were reconstituted with 80 µL of 30% methanol: 70% (0.1% formic acid) and transferred into LC-MS vials for analysis.

Urine specimens (200 µL) were added with ACN (400 µL), vortex-mixed for 5 min, and centrifuged at 4000 rpm for 7 min. The supernatant was collected and evaporated to dryness using a nitrogen evaporator at 60 °C. Thereafter, they were reconstituted with 80 µL of 10% methanol: 90% (0.1% formic acid) and transferred into LC-MS vials for analysis.

Faeces specimens were weighed, ultrapure water (at 8 times the weighed samples) was added, and samples were homogenised. The homogenised faeces samples (200 µL) were added with ACN (400 µL), vortex-mixed for 5 min, and centrifuged at 4000 rpm for 7 min. The supernatant was collected and evaporated to dryness using a nitrogen evaporator at 60 °C. Thereafter, they were reconstituted with 80 µL of 5% methanol: 95% (0.1% formic acid) and transferred into LC-MS vials for analysis.

Tissue specimens (kidneys and livers) were blotted with filter paper, weighed, and minced. Ultrapure water (at 3 times the weighed tissue samples) were added and samples were homogenised. The homogenised tissue specimens (200 µL) were added with ACN (400 µL), vortex-mixed for 5 min, and centrifuged at 4000 rpm for 7 min. The supernatant was collected and evaporated to dryness using a nitrogen evaporator at 60 °C. Thereafter, they were reconstituted with 80 µL of 5% methanol: 95% (0.1% formic acid) and transferred into LC-MS vials for analysis.

### 2.6. LC-MS/MS Analysis

Eurycomanone detection in the pure eurycomanone experiments was done via the LC-MS/MS system consisting of an Agilent 1260 Infinity HPLC system and an AB SCIEX API 4000™ Triple-Quadrupole mass spectrometer equipped with TurboIonSpray^®^ probe and atmospheric pressure chemical ionization (APCI) (AB Sciex LLC, Framingham, MA, USA). Electrospray ionization (ESI) was performed in positive ion mode. The analytical column was Agilent Zorbax Eclipse-C18 (4.6 mm I.D. × 150 mm, 3.5 µm). The mobile phase consisted of 0.1% formic acid (solvent A) and 90% ACN with 0.1% formic acid (solvent B). The flow rate was set at 1.2 mL/min and the injection volume was 50 µL with the run time of 3.5 min. The mass transitions, monitored using multiple-reaction monitoring (MRM) detections, were 409 → 221.1/143.1 for eurycomanone. Eurycomanone detection in the SWE experiments were done via an LC-MS system consisting of Agilent 1100 series HPLC and mass spectrometer detector system with APCI-ESI ionization mode (Agilent Technologies, Santa Clara, CA, USA).

For plasma specimens, the analytical column used was Agilent Zorbax Eclipse XDB-Phenyl (2.1 mm I.D. × 50 mm, 5 µm). The mobile phase consisted of 35% methanol: 65% (0.1% formic acid). The flow rate was set at 0.2 mL/min and the injection volume was 2 µL with the run time of 5 min. For urine samples, the analytical column was Agilent Zorbax Eclipse XDB-Phenyl (2.1 mm I.D. × 50 mm, 3.5 µm). The mobile phase consisted of 10% methanol: 90% (0.1% formic acid). The flow rate was set at 0.2 mL/min and the injection volume was 2 µL with the run time of 5 min.

For faeces, kidney, and liver specimens, the analytical column used was Agilent Zorbax Eclipse XDB-Phenyl (2.1 mm I.D. × 50 mm, 3.5 µm). The mobile phase consisted of 5% methanol: 95% (0.1% formic acid). The flow rate was set at 0.2 mL/min and the injection volume was 2 µL with the run time of 6 min. The identification of eurycomanone and internal standard for plasma matrix were achieved by comparing the ion sets in selected ion monitoring (SIM) for eurycomanone with sodium adduct (*m*/*z* 431.0) and metronidazole (172.0 g/mol) in samples with the standard solution, at a similar retention time using LC-MS analysis. The concentration of the specimens analysed were determined by using an inverse prediction method for the best-fit regression curve using the weighted least-square (WLS) of $\frac{1}{x^{2}}$, obtained from the standard concentrations.

In the analytical run, each standard in the calibration curve for each matrix was checked for the accuracy to be in the range of 80–120% for the lower limit of quantification (LLOQ) concentration and 85–115% for the remaining standards. Each quality control (QC) concentration was also checked for the accuracy to be in the range of 80–120% for the LLOQ concentration and 85–115% for the remaining QC samples. Sample matrices from the control group (blank samples) were used in the preparation of the calibration curve for determining eurycomanone concentrations. The pharmacokinetic analysis was performed using the non-compartmental analysis of Phoenix^TM^ WinNolin® software (version 1.3, Certara, L.P., Princeton, NJ, USA).

### 2.7. Aqueous Solubility, Lipophilicity, and Chemical Stability of Eurycomanone

The aqueous stability was estimated by adding 10 µL of eurycomanone stock solution (10 mM) to 490 µL of aqueous buffer at pH 5.4, pH 7.4 and DMSO in triplicates in deep-well plates. Three standards (propanolol, estriol, and tamoxifen) at the same concentration were also added in the deep-well plates. The plate was kept on a shaker (300 rpm) at room temperature for 16 h. The plate was then centrifuged at 25 °C, 4000 rpm for 25 min. The supernatant was transferred and analysed using LC-MS/MS. The aqueous solubility was calculated using the following formula:

$$\mathrm{Aqueous}\;\mathrm{stability}=\frac{200\;\mu M\times \mathrm{Peak}\;\mathrm{area}\;1}{\mathrm{Peak}\;\mathrm{area}\;2}$$

Peak area 1 = peak area of the compound in 2% DMSO at the different pH levels.

Peak area 2 = peak area of the compound in 100% DMSO.

The lipophilicity of eurycomanone was estimated using the shake flask method with octanol and buffer (pH 7.4). Eurycomanone (5 µL) at 5 mM concentration is added to octanol (495 µL) and DMSO (495 µL) and also into the aqueous and octanol phase in a 1:1 ratio (247.4 µL of octanol phase and 247.5 µL of aqueous phase). The octanol and aqueous phases were prepared using a saturation process, where equal amounts of D-PBS (pH 7.4) and octanol were saturated (300 rpm, room temperature) for 24 h before being separated and kept at room temperature. The prepared mixtures were vortexed and kept on the shaker (300 rpm) at room temperature for 16 h. The mixtures were allowed to stand for 30 min, and 200 µL of solution was transferred and sent for HPLC analysis. The analyses were conducted along with propranolol, estriol, metoprolol tartrate, and diethylstil bestrol (5 mM) as standards and were conducted in triplicates. 

The chemical stability of eurycomanone was estimated in gastric simulated fluid (GSF), pH 2; fasted simulated intestinal fluid (FASSIF), pH 6.5; fed simulated intestinal fluid (FESSIF), pH 5.0; and in 150 mM NaHCO_3_ at pH 9.2. Eurycomanone (3 µL) at 5 mM was added to 297 µL of the respective buffers. The mixture (100 µL) was added to 100 µL of ACN at time points 1 and 3 h and sent for LC-MS/MS analysis. The test was done in triplicates with propranolol and erythromycin (5 mM) as standards.

### 2.8. Permeability Assay

#### 2.8.1. Parallel Artificial Membrane Permeability Assay (PAMPA)

Eurycomanone was added to Pion buffer (Pion Inc., Billerica, MA, USA) pH 7.4 to make a 10 µM working solution. Five microlitres of the lipid mix was applied on the membrane and allowed to dry for 10 min. The working solution (200 µL) was added to the donor plate and Pion buffer (200 µL) were added to the receiver plate. The plate was kept in a moist chamber and left for 18 h at room temperature. The solution from both the donor and receiver plate and the working solution was analysed using LC-MS/MS. This test was carried out in triplicates using propranolol, atenolol, and carbamazepine as standards. The test concentration for eurycomanone is 25 µM.

#### 2.8.2. Caco-2 Cell Permeability Assay

The Caco-2 assay was carried out after seeding the cells onto the membrane to form a confluent monolayer in 21 days. The media is removed from apical (A) and basolateral (B) chambers and the membrane is washed with HBSS buffer. Eurycomanone (10 µM, 200 µL) was added to the A compartment (which represents the intestinal lumen), and buffer (300 µL) was added to the B compartment (representing the blood), and vice versa. The plate was kept in a shaking incubator (37 °C) for 1 h. The amount of eurycomanone that had permeated across the cells was measured by LC-MS/MS, and the apparent permeability (P_app_) values and efflux ratio were calculated. The expression of P-glycoprotein (P-gp) by differentiated Caco-2 cells was confirmed by measuring the P_app_ value and efflux ratio of vinblastine with and without the presence of a competing P-gp substrate, cyclosporine A. Eurycomanone was also measured with the presence of cyclosporine A to investigate whether eurycomanone may be transported via P-gp transporters.

### 2.9. Liver Microsome Metabolic Stability Assay

The liver microsome stability assay of eurycomanone was estimated using phase I and phase II enzymes in five types of microsomes (mice, rat, dog, monkey, and human). Eurycomanone at 1 mM (20 µL), microsomes at 0.3 mg/mL (20 µL), and 10 mg/mL of alamethicin in 1:1 DMSO and methanol (20 µL) were preincubated at 37 °C for 10 min. The cofactor, 5 mM UDPGA (20 µL), and 1 mM NADPH (20 µL) were added to make the final assay volume of 100 µL. At timepoints 0, 15, 30, 45, 60, and 90 min, 100 µL of the reaction mixture is taken out and added to the stop solution. The supernatants (10,000 rpm, 10 min, 4 °C) were collected and analysed using LC-MS/MS. The standards used for mice and rat liver microsomes were propranolol and dasatinib. The standards used with the dog and monkey liver microsomes were propranolol and midazolam, while the standards used with the human liver microsomes were propranolol and terfinadine.

### 2.10. Plasma Stability

The plasma stability was estimated by preparing 5 µM of eurycomanone in 600 µL plasma in triplicates. The plasma stability of eurycomanone was determined in five types of plasma (mice, rat, dog, monkey, and human). The reaction was kept at 37 °C and ACN (100 µL) was added at timepoints 0, 30 min, and 1, 2, and 4 h to the reaction mixture (100 µL). The supernatant (10,000 rpm, 10 min, 4 °C) was collected and analysed by LC-MS/MS. The standards (propantheline bromide and enalapril) were analysed in triplicates.

### 2.11. Plasma Protein Binding Assay

The ability of eurycomanone to bind plasma protein was analysed in five types of plasma (mice, rat, dog, monkey, and human). Each type of plasma was spiked with eurycomanone (1 mM) to make the 10 µM test concentration of eurycomanone in the plasma. Equilibrium dialysis buffer (500 µL) at pH 7.4 was added to the right chamber in rapid equilibrium dialysis (RED), while the spiked plasma (300 µL) was added to the left chamber. The Teflon base plate was covered with aluminium foil and left on the shaking incubator at 37 °C for 4 h. The matrix was balanced by mixing 100 µL of the equilibrium buffer with the spiked plasma from the left chamber in RED before adding the stop solution (350 µL) to precipitate out the protein. The supernatant (10,000 rpm, 10 min, 4 °C) was collected and analysed using LC-MS/MS. The standards (propranolol, warfarin, and acebutalol) were analysed in duplicates.

### 2.12. Statistical Analysis

The statistical analysis was performed using one-way or two-way ANOVA with either Dunnett or Tukey analysis chosen as the post-hoc analysis (GraphPad Prism, version 7.00 for Windows, GraphPad Software, La Jolla, CA, USA). Statistical significant differences were recognised at *p* ≤ 0.05.

## 3. Results

### 3.1. Pharmacokinetics of Eurycomanone in Mice

**Figure 1:**
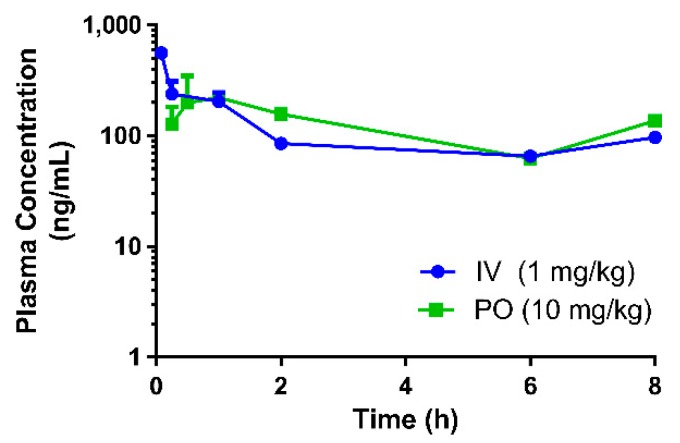
Pharmacokinetic profile of eurycomanone when administered as its pure compound in mice. (IV = intravenous route, PO = oral route)

A summary of the PK parameters in mice obtained following an intravenous and oral dose of eurycomanone is shown in Table 1. The plots for the mean plasma concentration of eurycomanone over time are shown in Figure 1. Following intravenous administration to mice, plasma clearance was moderate (3.85 L/h/kg) for eurycomanone. The volume of distribution was small (1.51 L/kg) for eurycomanone, with the elimination half-life of 0.30 h. Following oral administration, the absorption was moderate, where the T_max_ was observed at 2 h post-dose for the eurycomanone (C_max_ 334.7 ng/mL). The bioavailability of eurycomanone in mice was moderately high at 54.9% compared to rats. At 8 h post-dosing, the concentration of eurycomanone increased slightly in mice for both administration routes (Figure 1), which may be an indication of the enterohepatic circulation of eurycomanone in its administered form.

### 3.2. Pharmacokinetics of Eurycomanone and SWE in Rats

**Figure 2:**
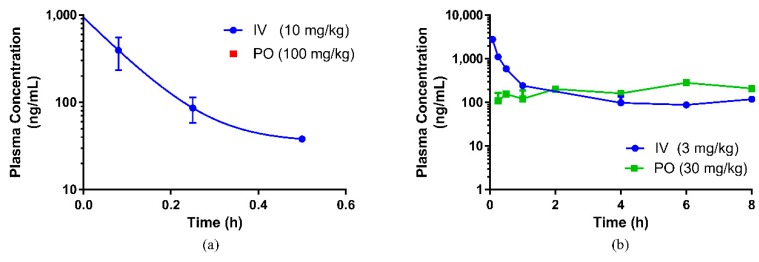
Pharmacokinetic profile of (**a**) standardized water extract (SWE) of *E. longifolia* and (**b**) eurycomanone compound, respectively, in rats. (IV = intravenous route, PO = oral route).

A summary of the PK parameters in rats obtained following an intravenous and oral dose of eurycomanone and the SWE is shown in Table 2. The plots for the mean plasma concentration of eurycomanone over time are shown in Figure 2a,b. Following intravenous administration to rats, the plasma clearance was moderate (2.74 L/h/kg) for eurycomanone in the compound form and low (0.76 L/h/kg) for eurycomanone in the SWE. The volume of distribution was low (0.95 L/kg) for eurycomanone and lower (0.12 L/kg) for the SWE, while their elimination half-lives were 0.30 and 0.12 h, respectively. Following oral administration, the absorption was moderate, where the T_max_ was observed at 2 h post-dose for the eurycomanone (C_max_ 238.3 ng/mL). The bioavailability of eurycomanone in rats was calculated to be low at 11.8%. At 6 and 8 h post-dosing of eurycomanone in the compound form, the concentration of eurycomanone increased slightly for the oral and intravenous routes, respectively (Figure 2). This may indicate that eurycomanone is recirculated via enterohepatic circulation in its unchanged administered form.

When SWE was orally administered, the plasma concentration of eurycomanone was undetectable in the plasma and it was mainly eliminated via faecal excretion. However, eurycomanone was detected in the liver samples at 47.6 ng/mL (intravenous) and 104.7 ng/mL (oral) at 24 h post-administration of SWE, indicating that a minimal amount of eurycomanone may have been absorbed, though not detectable. Intravenous administration of SWE showed that eurycomanone remains in the plasma up until 30 min and is mainly eliminated via urinary excretion.

### 3.3. Eurycomanone Aqueous Solubility, Lipophilicity, and Chemical Stability

**Figure 3:**
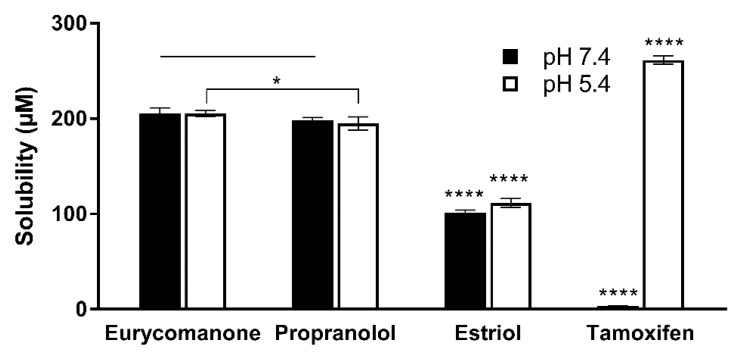
Aqueous solubility of eurycomanone, propranolol, estriol, and tamoxifen at pH 7.4 and pH 5.4. The values were plotted based on the mean ± standard deviation. **** denotes significant difference when compared to eurycomanone (*p* ≤ 0.0001) at the two pH values. * denotes significance difference between propranolol and eurycomanone at pH 5.4 (*p* ≤ 0.05). Line over bars indicates no significant difference when compared to eurycomanone (*p* ≥ 0.05).

**Figure 4:**
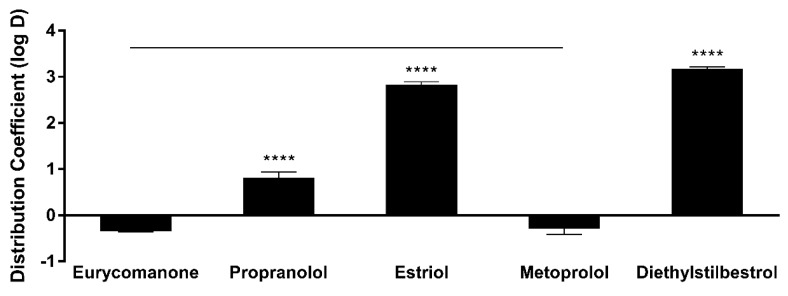
Lipophilicity properties of eurycomanone, propranolol, estriol, metoprolol, and diesthylstilbestrol. The distribution coefficient (log D) value for eurycomanone was −0.35. All standards showed acceptable log D values, respectively. The values were plotted based on the mean ± standard deviation. **** denotes significant difference when compared to eurycomanone (*p* ≤ 0.0001). The log D value of eurycomanone is comparable to that of metoprolol. Line over bars indicates no significant difference between metoprolol and eurycomanone (*p* ≥ 0.05).

**Figure 5:**
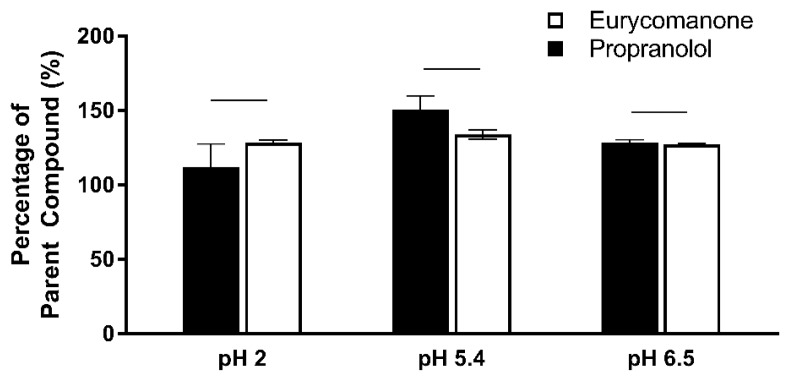
Eurycomanone showed high stability at pH 2, pH 5, and pH 6.5 and is comparable to the stability of propranolol. The values were plotted based on the mean ± standard deviation. There were no significant differences between propranolol and eurycomanone at all the pH levels tested. Line over bars indicates no significant difference when compared to eurycomanone (*p* ≥ 0.05).

Eurycomanone was highly soluble at pH 5.4 (205.5 µM) and 7.4 (205.4 µM) and was comparable to propranolol (Figure 3). Estriol, which has moderate solubility at both pHs, and tamoxifen, which was insoluble at pH 7.4 and highly soluble in pH 5.4, were used as internal controls. Due to this highly aqueous nature of eurycomanone, it is expected that its distribution coefficient log D at pH 7.4 is low (−0.35). Propranolol (0.82), estriol (2.82), metoprolol (−0.29), and diethylstilbestrol (3.18) were assayed together as internal controls as they have a wide range of log D values (Figure 4). The chemical stability of eurycomanone at the varying pH levels, mimicking changing pH environments in physiological conditions, i.e., using GSF (pH 2), FESIIF (pH 5.0), and FASSIF (pH 6.5), were also carried out. The percentage of eurycomanone remaining after 3 h of incubation showed that eurycomanone is chemically stable across all pH values tested (Figure 5).

### 3.4. Eurycomanone Permeability

**Figure 6:**
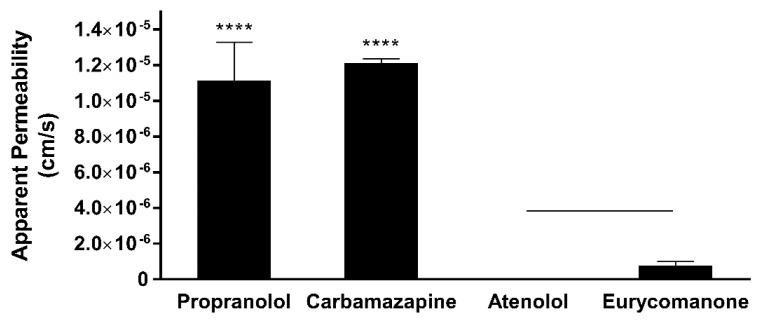
Eurycomanone showed low permeability as compared to propranolol and carbamazepine when assayed using the parallel artificial membrane permeability assay (PAMPA). The values were plotted based on the mean ± standard deviation. **** denotes significant difference when compared to eurycomanone (*p* ≤ 0.0001). Line over bars indicates no significant difference between atenolol and eurycomanone (*p* ≥ 0.05).

The rapid assessment of the absorption ability of eurycomanone was conducted via the PAMPA. Eurycomanone’s permeation through this artificial membrane is low at 0.78 × 10^−6^ cm/s (Figure 6). As the PAMPA system lacks some similarity to natural membranes, i.e., lack of pores and proteins responsible for active transport such as P-gp protein, eurycomanone was also evaluated in a Caco-2 cell monolayer (Table 3). However, eurycomanone showed low permeability in Caco-2 cells at 0.45 × 10^−6^ cm/s (A to B direction) and 0.73 × 10^−6^ cm/s (B to A direction), similar to its permeation in the artificial membrane. Eurycomanone was not a substrate for efflux transporter as the efflux ratio is less than 2. Incubation of eurycomanone with cyclosporine A (a P-gp substrate) showed no changes and no competition with eurycomanone. Alongside eurycomanone, vinblastine, which is also a P-gp substrate, was also tested to ensure the Caco-2 cells were expressing P-gp transporters.

### 3.5. Liver Microsome Metabolic Stability

Eurycomanone was relatively stable in rat liver microsomes and hepatocytes and in human liver microsomes, with the half-life of more than 90 min (Table 4). The intrinsic clearances (Cl_int_) calculated from the rate of metabolism of eurycomanone in the liver microsomal incubations were high for rat (83.16 mL/min/kg) and slightly lower in human microsomes (52.39 mL/min/kg). Intrinsic clearance in the rat liver hepatocytes was also slow at 0.67 µL/min/million cells. These results suggest that eurycomanone is not a species-specific substrate and that is may be highly metabolised in the liver.

### 3.6. Plasma Stability and Plasma Protein Binding

**Figure 7:**
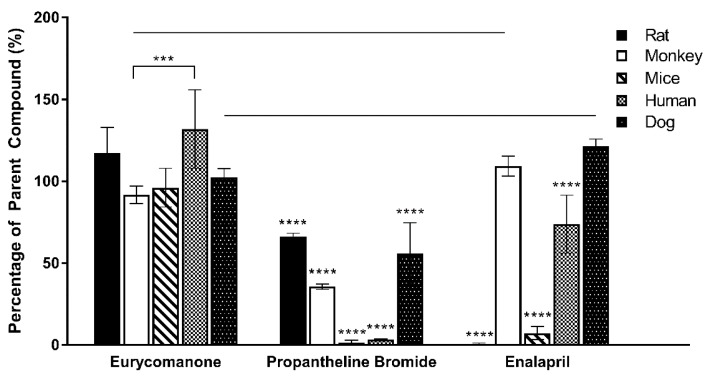
Eurycomanone’s stability in rat, monkey, mice, human, and dog plasma as compared to propantheline bromide and enalapril. The values were plotted based on the mean ± standard deviation. **** denotes significant difference when compared to eurycomanone (*p* ≤ 0.0001) in the five species tested. *** denotes significance difference (*p* ≤ 0.001) in eurycomanone’s stability between species monkey and human. Line over bars indicates no significant difference when compared to eurycomanone in species monkey and dog for enalapril (*p* ≥ 0.05).

**Figure 8:**
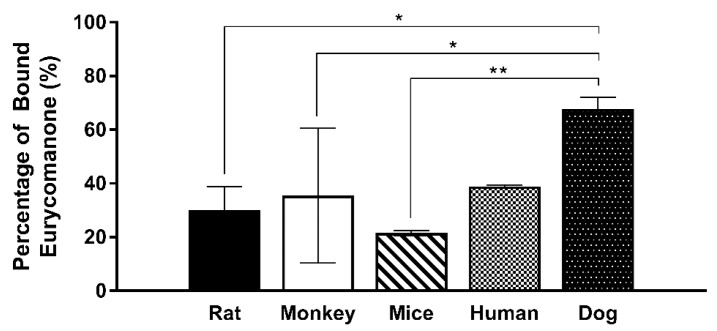
Eurycomanone plasma protein binding in rat, monkey, mice, human, and dog plasma. The values were plotted based on the mean ± standard deviation. ** and * denote significant difference when compared to across species (*p* ≤ 0.01) and (*p* ≤ 0.05) respectively. No significant difference was found between the rest of the species tested (*p* ≥ 0.05) (not indicated on graph).

Eurycomanone was highly stable in the human, rat, and dog plasma, while stability was slightly lower in the monkey and mice plasma (Figure 7). The varied species used in the plasma profiling were used to assess possible differences in eurycomanone’s stability as exhibited by other compounds such as propantheline bromide and enalapril. However, eurycomanone were shown to be stable in all the species tested. From the plasma protein binding study (Figure 8), it was found that the percentages of bound eurycomanone were 30.1% (rat), 35.4% (monkey), 21.6% (mice), 38.9% (human), and 67.8% (dog). Apart from in the dog plasma, eurycomanone exhibited a low plasma binding ability in all the other species’ plasma, with a high fraction of unbound eurycomanone (61.1–78.4%) with free access to its target sites.

## 4. Discussion

Eurycomanone has been evaluated for its biological activities via various in vitro methods [17,20,24,25,26,27], while the extract form was mainly used in various in vivo tests [28,29,30,31,32]. From this study, the concentration of the pure eurycomanone in rat plasma was at its maximum (C_max_ 238.3 ng/mL) at 2 h post oral administration, and the bioavailability for the eurycomanone compound was low (11.8%). The findings in this study with regard to eurycomanone’s bioavailability was similar to findings by Low et al. [22], where it was reported that the bioavailability of the orally administered fortified eurycomanone in *E. longifolia* extract was 10.5% and the C_max_ was found to be 330 ng/mL. There was no notable difference in terms of eurycomanone’s bioavailability and maximum concentration when using a pure compound as in the current study and when compared to using a fortified extract as used by Low et al. [22]. Rehman and his coworkers, in their investigation, found the C_max_ to be lower at 40.43 ng/mL for the pure eurycomanone and 9.90 ng/mL for eurycomanone in *E. longifolia* extract [7]. In this study, interspecies variation in the bioavailability of eurycomanone was noted. The pure compound was found to be more bioavailable (higher absorption) in mice (54.9%) than in rats, and the C_max_ (335 ng/mL) in mice was slightly higher than the levels found in rats. This also indicates that eurycomanone may behave differently in different species and the PK values may not be able to be directly extrapolated from rodents to humans. This species-dependent characteristic of eurycomanone behaviour in rodents may be due to species differences in the metabolism and disposition of eurycomanone, as there was a variation in the absorption, distribution, and metabolism of eurycomanone, in which they were faster in mice than in rats. Two peaks were observed in the PK profiles in both the oral and intravenous profiles. The presence of these secondary peaks may indicate the occurrence of enterohepatic circulation of eurycomanone [33]; however, this must be further investigated in a separate study. Ma et al. suggest that the secondary peak of eurycomanone may be caused by eurycomanone’s activity as a muscle relaxant, which caused delayed gastric emptying and hence delayed intestinal absorption, resulting in the secondary peak in the profile [23]. In the present study, the plasma concentration–time profile was only conducted for 8 h, which limits the window of analysis to confirm the second peak. 

Previously, Zakaria et al. reported eurycomanone activity in vivo, where eurycomanone was orally administered at 6 mg/kg and 17 mg/kg to mice. At the dose of 17 mg/kg, tumour suppression ability was demonstrated in nude mice with HepG2 cell-induced tumours, which showed that there was sufficient or better bioavailability to exert the reported efficacy [32]. However, in relation to previous in vitro studies, the C_max_ value obtained in the current study was much lower than those shown in previous studies of eurycomanone and *E. longifolia* extracts (in the range of micrograms to miligrams) [17,20,24,25,26,27]. The reported efficacy via in vitro models may not accurately represent eurycomanone’s level in vivo. Stability in plasma was measured via predetermined time incubation (0 and 30 min and 1, 2, 4 h) of eurycomanone and it was found to be highly stable; nevertheless, eurycomanone’s ability to bind to proteins present in the plasma may affect its availability to its target site of action. However, the plasma protein binding in rats indicates that a low percentage of eurycomanone (30%) is bound to plasma proteins. In other words, the remaining 70% unbound fraction of eurycomanone is freely available and can possibly access the target sites [34]. 

The low absorption of eurycomanone in vivo correlates with findings from in vitro studies, namely the lipophilicity, solubility, and permeability assessment of eurycomanone. The lipophilicity of eurycomanone was very low and its solubility was high, which influences its permeation rate via lipid membranes as well as cell membranes such as of Caco-2 cells. The low log D value determined in this study indicates the extent to which eurycomanone was likely to stay in the aqueous environment instead of dissociating into the hydrophobic octanol. This relates to eurycomanone being highly soluble and its low permeation when assayed using the PAMPA method. When eurycomanone’s permeability was assessed using the Caco-2 cells, the results also indicated that a low amount of eurycomanone permeated the cell monolayer. However, this may be due to the condition where eurycomanone, which was not lipophilic enough to be absorbed, accumulates on the cell monolayer, introducing a sink condition [35]. This accumulation then draws the compound to cross the membrane, which may not be due to the compound’s innate ability to cross the membrane freely. 

The ability of the compound to cross the biological membrane without the use of transporters is possible when the compound of interest is in its uncharged state. Eurycomanone is predicted to have an acid dissociation constant (pK_a_) value of 11 [36]. The pH values of the environments in the stomach and small intestine vary in the different sections and depending on the fasted or fed state. The pH range in the stomach in its fasted state is between 1.4 and 2.1; while in its fed state, it is in between 4.3 and 5.4. In the small intestine, the pH values of the different segments in their fasted state are 4.9–6.4 (duodenum); 4.4–6.6 (jejunum), and 6.5–7.4 (ileum); while in their fed state, the pH values are 4.2–6.1 (duodenum); 5.2–6.2 (jejunum), and 6.8–7.5 (ileum) [37]. The changing pH values then affect the ionic state of eurycomanone. The predicted pK_a_ for eurycomanone was high, which means that in a lower pH environment, eurycomanone exists in its ionised form. This then prevents eurycomanone from crossing the membrane barriers passively. 

As determined in the Caco-2 permeability assessment, eurycomanone was not an active transporter P-gp substrate. The chemical stability assay conducted using the fasted and fed state simulative buffer system indicates that eurycomanone was stable; however, its ionic state could not be confirmed. The plasma stability and liver metabolic stability study also showed eurycomanone to be stable in the different matrices, indicating that eurycomanone may stay intact and survive first-pass metabolism in vivo. This study hypothesised that when eurycomanone was administered orally, only a small amount crosses the membrane to enter the blood stream, while when it was administered intravenously, only a small amount of eurycomanone permeates into tissues, while most remained in the blood in its ionised form. The small amount of eurycomanone which may have been absorbed (as a low amount was detected in liver tissues) could still potentially exert the desired effects of eurycomanone or *E. longifolia* extract in general.

## 5. Conclusions

In conclusion, the results indicate that eurycomanone was a very polar compound with high stability at different pHs, in the plasma and in liver microsome. No major degradation of the compound in the gastrointestinal tract and blood plasma was noted and eurycomanone also exhibited low plasma protein binding ability. Despite the favourable properties of eurycomanone, its low permeability hinders its absorption, hence its bioavailability in in vivo models. The low absorptive value of eurycomanone may indicate the need to use eurycomanone at lower concentrations in assays aimed at finding its efficacy. The adoption of eurycomanone as the bioactive marker for *E. longifolia* may be considered with further evaluations of eurycomanone in in vivo models.

## Figures and Tables

**Table 1:** Summary of pharmacokinetic parameters following an intravenous and oral dose of eurycomanone to mice.

Parameters (Mice)	Units	Eurycomanone	
		1 mg/kg	10 mg/kg
		Intravenous	Oral
AUC_0–8_	h∙ng/mL	221.0	1213
Plasma Clearance (CL)	L/h/kg	3.85	NA
Volume of Distribution at Steady State (V_ss_)	L/kg	1.51	NA
Half-life (t_1/2_)	h	0.30	NA
Observed T_max_	h	NA	2.0
Observed C_max_	ng/mL	NA	334.7
Bioavailability (F)	%	NA	54.9

NA: Not applicable, AUC: Area under the curve, T_max_: time taken to reach the maximum concentration, C_max_: maximum concentration of the compound achieved in the plasma.

**Table 2:** Summary of pharmacokinetic parameters following an intravenous and oral dose of eurycomanone and SWE to rats.

Parameters (Rats)	Units	Eurycomanone		SWE	
		3 mg/kg	30 mg/kg	10 mg/kg	100 mg/kg
		Intravenous	Oral	Intravenous	Oral
AUC_0–8_	h∙ng/mL	970.0	1149.0	100.8	ND
Plasma Clearance (CL)	L/h/kg	2.74	NA	0.76	NA
Volume of Distribution at Steady State (V_ss_)	L/kg	0.95	NA	0.12	NA
Half-life (t_1/2_)	h	0.30	NA	0.11	NA
Observed T_max_	h	NA	2.0	NA	ND
Observed C_max_	ng/mL	NA	238.3	NA	ND
Bioavailability (F)	%	NA	11.8	NA	NC
Urinary excretion 24 h post-dose	%	NA	NA	22.52	5.02
Fecal excretion 24 h post-dose	%	NA	NA	0.56	33.02
Liver accumulation	ng/mL	NA	NA	47.6	104.7
Kidney accumulation	ng/mL	NA	NA	NA	NA

NA: Not applicable, ND: Not detected, NC: Not calculated, AUC: Area under the curve, T_max_: time taken to reach the maximum concentration, C_max_: maximum concentration of the compound achieved in the plasma.

**Table 3:** Apparent permeability of eurycomanone assayed via the Caco-2 permeability assay.

Tested Compounds	Direction	P_app_ (× 10^−^^6^ cm/s)	Efflux Ratio
Eurycomanone	A to B	0.45	1.62
	B to A	0.73	
Eurycomanone + Cyclosporine A	A to B	1.20	1.13
	B to A	1.35	
Vinblastine	A to B	3.76	14.72
	B to A	55.34	
Vinblastine + Cyclosporine A	A to B	16.71	2.39
	B to A	40.00	

P_app_: Apparent permeability.

**Table 4:** Rat liver microsomes and hepatocytes and human liver microsome stability of eurycomanone.

Parameters	Units	Rat Liver Microsomes	Rat Liver Hepatocytes	Human Liver Microsomes
Half-life (t_1/2_)	h	>1.5	>1.5	>1.5
Intrinsic clearance (Cl_int_)	mL/min/kg	83.16	NA	52.39
Intrinsic clearance (Cl_int_)	µL/min/million cells	NA	0.67	NA

NA: Not applicable.
